# The Anti-neuroinflammatory Activity of Tectorigenin Pretreatment via Downregulated NF-κB and ERK/JNK Pathways in BV-2 Microglial and Microglia Inactivation in Mice With Lipopolysaccharide

**DOI:** 10.3389/fphar.2018.00462

**Published:** 2018-05-09

**Authors:** Hye-Sun Lim, Yu Jin Kim, Bu-Yeo Kim, Gunhyuk Park, Soo-Jin Jeong

**Affiliations:** ^1^Herbal Medicine Research Division, Korea Institute of Oriental Medicine, Daejeon, South Korea; ^2^College of Pharmacy, Chungnam National University, Daejeon, South Korea; ^3^Ektos Industries Co. Ltd., Daejeon, South Korea; ^4^Korean Medicine Life Science, University of Science & Technology, Daejeon, South Korea

**Keywords:** tectorigenin, neuroinflammation, microglia, mitogen-activated protein kinases, nuclear factor-κB

## Abstract

The activation of microglia is decisively involved with the neurodegeneration observed in many neuroinflammatory pathologies, such as multiple sclerosis, Parkinson’s disease, and Alzheimer’s disease. Tectorigenin (TEC) is an isoflavone isolated from various medicinal plants, such as *Pueraria thunbergiana* Benth, *Belamcanda chinensis*, and *Iris unguicularis*. In the present study, the neuroinflammatory effects of TEC were evaluated in both lipopolysaccharide (LPS)-treated BV-2 microglial and mouse models. TEC remarkably inhibited reactive oxygen species (ROS) generation. TEC also inhibits the production and expression of nitric oxide (NO), prostaglandin E_2_ (PGE_2_), tumor necrosis factor-α (TNF-α), and interleukin-6 (IL-6) in LPS-stimulated BV-2 cells. In addition, TEC suppressed the LPS-induced activation of nuclear factor-κB (NF-κB), phosphorylation of extracellular signal-regulated kinase (ERK), and c-Jun N-terminal kinase (JNK) to regulate the inflammatory mediators, such as inducible NO synthase (iNOS), cyclooxygenase-2 (COX-2), TNF-α, and IL-6. These results indicate that TEC may inhibit neuronal inflammation through the downregulation of inflammatory mediators, including iNOS, COX-2, TNF-α, and IL-6 by suppressing NF-κB/ERK/JNK-related signaling pathways. Furthermore, cotreatment with TEC and ERK inhibitor SCH772984 or JNK inhibitor SP600125 suppressed the overproduction of LPS-induced NO production in BV-2 cells. Consistent with the results of *in vitro* experiments, an LPS-induced brain inflammation mouse model, administration of TEC effectively decrease the levels of malondialdehyde, iNOS in hippocampus, and prevented increases in the levels of TNF-α and IL-6 in the serum. TEC showed marked attenuation of microglial activation. Finally, TEC inhibited protein expression of toll-like receptor 4 and myeloid differentiation factor 88 in LPS-activated BV-2 microglia and mouse models. Taken altogether, the cumulative findings suggested that TEC holds the potential to develop as a neuroprotective drug for the intervention of neuroinflammatory disorders.

## Introduction

Neuroinflammation is characterized by microglial activation and has been closely associated with the pathogenesis of Alzheimer’s disease (AD), as well as several other neurodegenerative disorders, including Parkinson’s disease (Liu and Hong, 2003). Microglia is macrophages in the brain that play a role in immune surveillance and host defense under normal conditions (Prasad et al., 2015). Neuronal cell death causes microglial activation through the release of various signaling molecules such as nuclear factor (NF)-κB, mitogen-activated protein kinases (MAPKs), and toll-like receptors (TLRs), suggesting that neuroinflammation occurs as a result of an ongoing neurodegenerative disease process (Kreutzberg, 1996; Kim et al., 2014). In addition, oxidative stress, which is characterized by the aberrant production of reactive oxygen species (ROS) and failure of the antioxidant defense system, has been associated with neurodegenerative diseases such as AD (Uttara et al., 2009; Gonzalez-Reyes et al., 2013). Cognitive deficits associated with AD are the result of increased susceptibility to oxidative stress and neuroinflammation (Uttara et al., 2009). Under pathological conditions, activated microglia can exhibit detrimental effects involving the overproduction of neurotoxic factors such as nitric oxide (NO), prostaglandin E_2_ (PGE_2_), and inflammatory cytokines, including tumor necrosis factor (TNF)-α and interleukin-6 (IL-6) (Amor et al., 2010). Therefore, inhibiting the aberrant activation of microglia may have therapeutic potential in the treatment of neuroinflammation-related neurodegenerative diseases.

Excessive release of inflammatory mediators may initiate the onset of neurodegeneration via various signaling pathways. Neuroinflammation is initiated by a variety of pathogens such as lipopolysaccharide (LPS), amyloid-β, bacteria, and viruses. These pathogenic stimuli activate NF-κB and MAPK signaling pathways. NF-κB, an important transcription factor that regulates the immune system, is translocated to the nucleus and promotes the expression of proinflammatory genes that process NF-κB-specific binding sites in their promoter regions. These molecules include inducible NO synthase (iNOS), cyclooxygenase-2 (COX-2), TNF-α, and IL-6, which in turn promote the production of the main proinflammatory mediators, NO, PGE_2_, TNF-α, and IL-6 (Eikelenboom and van Gool, 2004). There have been efforts to identify the upstream regulators of NF-κB activity, and ROS have been identified as potential candidates (Bonizzi et al., 1999). Additionally, phytocompounds inhibit the expression of iNOS, COX-2, and TNF-α in microglia by blocking aberrant NF-κB activation through inhibition of ROS generation (Korn et al., 2001; Surh et al., 2001; Camandola and Mattson, 2007). MAPKs are a major family of kinases associated with inflammatory processes. Moreover, LPS can activate NF-κB and MAPKs, including extracellular signal-regulated kinase (ERK), c-Jun N-terminal kinase (JNK), and p38 MAPK. These proteins then modulate cytokine production and the expression of proinflammatory enzymes, such as iNOS, COX-2, TNF-α, and IL-6 (Rao, 2001; Rajapakse et al., 2008). Therefore, NF-κB and MAPK are considered crucial elements in inflammatory processes and may act as effective targets for anti-neuroinflammatory therapy.

Toll-like receptor 4 (TLR4) is a cell-membrane receptor for LPS (Zhang et al., 2014). After recognizing LPS, TLR4 activates different signaling pathways, including myeloid differentiation factor 88 (MyD88)-dependent pathways. MyD88 is an adaptor protein which mediates signaling pathways for most TLRs, leading to activation of NF-κB and MAPKs. This subsequently drives the transcriptional abundance of proinflammatory signals (Seong et al., 2016).

**FIGURE 1:**
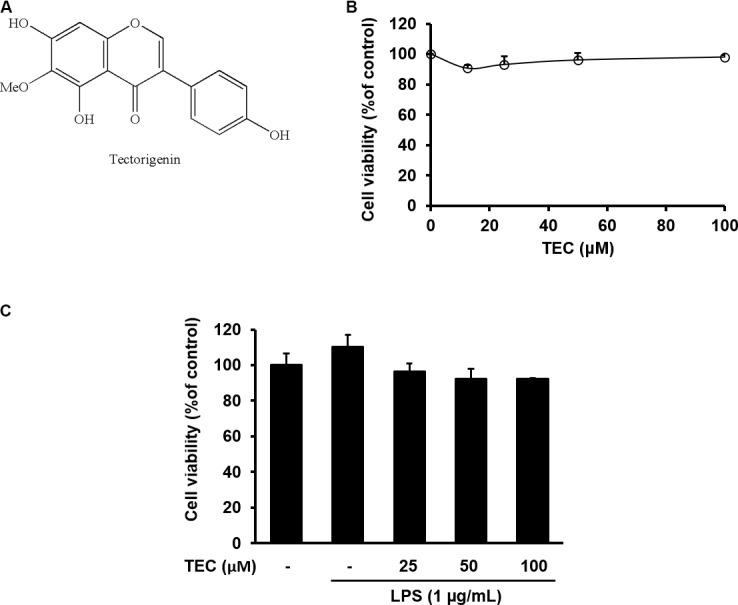
Cytotoxicity of TEC in BV-2 microglia. **(A)** Chemical structure of TEC. **(B)** Cells were seeded on 96-well plates and treated with TEC (0, 12.5, 25, 50, 100 μM) for 24 h. **(C)** Cell were pretreated TEC (25, 50, 100 μM) for 2 h followed by treatment with LPS (1 μg/mL) for 22 h. Cell viability was assessed using the CCK-8 assay. The results are expressed as the mean ± SEM of three independent experiments.

Tectorigenin (TEC, **Figure 1A**) is an active component of the traditional medicine isolated from *Pueraria thunbergiana* Benth, *Belamcanda chinensis*, and *Iris unguicularis*. TEC has been reported to exert pharmacological actions, including antitumor (Wani et al., 2013) and antibacterial effects (Oh et al., 2001), free radical neutralization (Kang et al., 2005), and selective estrogen receptor modulation (Shim et al., 2014). In addition, TEC was found to inhibit interferon-γ/LPS-induced inflammatory responses in murine macrophage RAW 264.7 cells (Pan et al., 2008). However, the precise biochemical mechanisms underlying the effects of TEC on neuroinflammation have yet to be clarified. Therefore, in the present study, we investigated the mechanisms underlying the inhibitory effects of TEC and its anti-inflammatory actions against LPS-stimulated inflammatory responses in murine BV-2 microglia and a mouse model of LPS-induced inflammation.

## Materials and Methods

### Cell Culture, Animals, and Drug Administration

Mouse microglia cell line BV-2 was maintained in Dulbecco’s modified Eagle’s medium (Hyclone/Thermo, Rockford, IL, United States) supplemented with 10% fetal bovine serum (Hyclone/Thermo) and 1% penicillin/streptomycin at 37°C under an atmosphere of 5% CO_2_. Male ICR mice (8 weeks old) were purchased from Dae Han Biolink (Eumseong, Korea) and housed in environmentally controlled and specific pathogen-free conditions (22°C, 12 h light/12 h dark cycle) and allowed water and standard food pellets *ad libitum*. All experimental procedures were conducted in accordance with the National Institutes of Health Guidelines for the Care and Use of Laboratory Animals and were approved by the Institutional Animal Care and Use Committee of the Korea Institute of Oriental Medicine (Approval No. 17-038). Animal handling was conducted in accordance with the dictates of the National Animal Welfare Law of Korea. After 1 week of acclimation in an animal care facility, mice were divided into 4 groups (*n* = 7/group): normal control, LPS group, and LPS + oral gavage of TEC at 5 or 10 mg/kg. An equal volume of saline vehicle was given to rats in the control and LPS groups. TEC was dissolved in saline and administered per os (p.o.) once per day for 5 days and LPS was dissolved in saline and injected intraperitoneally (5 mg/kg) 3 h after the final TEC administration (**Figure 6A**).

### Cell Viability Assay

Cell viability was evaluated by Cell Counting Kit (CCK-8; Dojindo, Kumamoto, Japan) according to the manufacturer’s protocol. In brief, cells were seeded on 96-well plates at a density of 3 × 10^4^ cells/well and treated with TEC (25, 50, or 100 μM; Chengdu Must Bio-Technology, Chengdu, China; CAS NO. 548-77-6; Purity, 95∼99%), and pretreated with TEC for 2 h prior to LPS (1 μg/mL; Sigma–Aldrich, St. Louis, MO, United States; CAT NO. L4391) treatment for 22 h. The CCK-8 reagent was added to each well and the mixture was incubated for 4 h. The absorbance was read at 450 nm using a Benchmark Plus microplate reader (Bio-Rad Laboratories, Hercules, CA, United States). Cell viability was calculated using the following equation: cell viability (%) = (mean absorbance in HSS-treated cells/mean absorbance in the untreated control) × 100.

### Nitric Oxide (NO) Assay

Nitric oxide concentrations in culture supernatants were determined by measuring nitrite, which is a major stable product of NO, using Griess reagent [1% sulfanilamide, 0.1% *N*-(1-naphthyl)-ethylenediamine dihydrochloride, and 2.5% H_3_PO_4_, Promega, Madison, WI, United States]. Cells (2 × 10^5^ cells/well) were pretreated with TEC for 2 h and treated with LPS (1 μg/mL) for an additional 22 h in 24-well plates. After collecting the culture supernatants, 50 μL culture medium was mixed with an equal volume of Griess reagent. Nitrite levels were determined using an enzyme-linked immunosorbent assay (ELISA) plate reader at 535 nm and nitrite concentrations were determined from a standard curve generated using sodium nitrite solutions.

### Measurement of PGE_2_, TNF-α, and IL-6 Production

The ELISA kit from Cayman Chemical (Ann Arbor, MI, United States) was used for the measurement of PGE_2_, and a kit from R&D Systems (Minneapolis, MN, United States) was used for the measurement of TNF-α and IL-6, according to the manufacturers’ protocols. Briefly, the supernatant of the cell culture was collected and centrifuged. Samples were applied to each well for ELISA. The concentration of each sample was calculated according to the standards provided in the kits.

### RNA Extraction and Reverse Transcription–Polymerase Chain Reaction Analysis

Total RNA was extracted from BV-2 cells using the TRIzol reagent (Invitrogen, Carlsbad, CA, United States). Equal amounts of RNA (1 μg) were reverse transcribed (RT) into cDNA using an iScript cDNA synthesis kit (Bio-Rad, Hercules, CA, United States) according to the manufacturer’s protocols. A polymerase chain reaction (PCR) was performed using the cDNA as a template and the following amplification conditions: 28 cycles of denaturing at 94°C for 30 s, annealing at 52°C for 1 min, and extension at 72°C for 90 s for iNOS and COX-2; 28 cycles of denaturing at 94°C for 30 s, annealing at 47°C for 1 min, and extension at 72°C for 90 s for TNF-α and IL-6; and 25 cycles of denaturing at 94°C for 30 s, annealing at 57°C for 1 min, and extension at 72°C for 90 s for β-actin. The following primers derived from the published cDNA sequences were used for the PCR amplifications: iNOS forward, 5′-CCTCCTCCACCCTAGCAAGT-3′ and reverse, 5′-CACCCAAAGTGCTTCAGTCA-3′; COX-2 forward, 5′-AAGACTTGCCAGGCTGAACT-3′ and reverse, 5′-CTTCTGCAGTCCAGGTTCAA-3′; TNF-α forward, 5′-TGGGTAGAGAATGGATGAAC-3′ and reverse, 5′-GCCGATTTGGTATCTCATAC-3′; IL-6 forward, 5′-AAGAGACTTCCATCCAGTTG-3′ and reverse, 5′-TCCAGGTAGCTATGGTACTC-3′; β-actin forward, 5′-TGTGATGGTGGGAATGGGTCAG-3′ and reverse, 5′-TTTGATGTCACGCACGATTTCC-3′. The PCR products were separated on a 1.5% agarose gel and visualized on an Azure C150 Gel Imaging Workstation (Azure Biosystems, Dublin, CA, United States). The relative expression levels of iNOS, COX-2, TNF-α, and IL6 mRNA were normalized to those of β-actin mRNA using a Chemi-Doc Band Analysis system (Bio-Rad Laboratories, Hercules, CA, United States).

### MDA Assay

The MDA level from LPS-induced brain was evaluated using TBARS kit (Cayman Chemical, Ann Arbor, MI, United States) according to the manufacturer’s instructions. Briefly, MDA reacted with thiobarbituric acid in the acidic high temperature and formed a red-complex TBARS. The absorbance of TBARS was determined at 532 nm. The assay procedure included with the kit was followed to obtain MDA concentrations, and results were caculated in μmoles MDA/g protein.

### Western Blot Analysis

TEC-treated cells or hippocampus tissues of mice were washed three times with PBS and lysed in lysis buffer (1% Triton X-100, 1% deoxycholate, and 0.1% NaN_3_) containing protease inhibitors (Roche Diagnostics, Mannheim, Germany). Nuclear and cytosol proteins were prepared using NE-PER nuclear and cytoplasmic extraction reagents (Pierce Biotechnology, Rockford, IL, United States) according to the manufacturer’s instructions. Subsequently, protein concentration was determined using a Bradford Protein Assay kit (Bio-Rad) according to the manufacturer’s instructions. Proteins (30 μg) were resolved by 10% sodium dodecyl sulfate–polyacrylamide gel electrophoresis (SDS–PAGE) and transferred to poly (vinylidene fluoride) membranes. The membranes were incubated with blocking buffer (5% skim milk in Tris-buffered saline containing 0.5% Tween 20 (TBST)), followed by overnight incubation at 4°C with the appropriate primary antibodies: NF-κB p65 and phosphorylated/total forms of p38 MAPK, ERK, JNK, iNOS (rabbit polyclonal antibodies, 1:1000 dilution; Cell Signaling Technology, Danvers, MA, United States), TLR4 and MyD88 (rabbit polyclonal antibodies, 1:1000 dilution; Santa Cruz Biotechnology, Dallas, TX, United States), and ionized calcium-binding adaptor molecule 1 (Iba-1) (rabbit antibodies, 1:1000 dilution; Wako Pure Chemical Industries, Osaka, Japan). GAPDH and nucleolin (rabbit polyclonal antibodies, 1:1000 dilution; Cell Signaling Technology) were used as internal controls for the whole cells and nuclear fraction, respectively. The membranes were washed three times with TBST and incubated with a 1:3000 dilution of a horseradish peroxidase-conjugated secondary antibody (Jackson ImmunoResearch, West Grove, PA, United States) for 1 h at room temperature. The membranes were washed again three times with TBST and developed using an enhanced chemiluminescence kit (Thermo Scientific, Rockford, IL, United States). Images of developed membranes were captured using an LAS 4000 mini luminescent image analyzer (GE Healthcare Bio-Sciences, Piscataway, NJ, United States). The relative expression levels were adjusted based on the expression of GAPDH or nucleolin as a control using ChemiDoc Band Analysis system (Bio-Rad Laboratories).

### Immunofluorescence Analysis

BV-2 cells were immunostained to detect NF-κB p65 protein expression. In brief, the cells were plated on glass coverslips and incubated overnight. After pretreatment with TEC (50 or 100 μM, 1 h) and LPS stimulation (1 μg/mL, 30 min), the cells were fixed in 4% paraformaldehyde for 30 min, permeabilized with PBS, and blocked with 1% bovine serum albumin (BSA) in PBS. Next, the cells were incubated with primary antibody (rabbit monoclonal antibody against the p65 subunit of NF-κB, Cell Signaling Technology) overnight at 4°C. The cells were washed with PBS and incubated with a fluorescently conjugated secondary antibody (Santa Cruz Biotechnology, Santa Cruz, CA, United States) for 1 h. The cells were counterstained by 4′,6-diamidino-2-phenylindole (DAPI). Then, mice were immediately anesthetized and perfused transcardially with 0.05 mol/L PBS, followed by cold 4% paraformaldehyde in 0.1 mol/L phosphate buffer. Brains were removed and postfixed in 0.1 mol/L phosphate buffer containing 4% paraformaldehyde overnight at 4°C and then immersed in a solution containing 30% sucrose in 0.05 mol/L PBS for cryoprotection. Serial 30 μm thick coronal sections were cut on a freezing microtome (Leica Instruments, Nussloch, Germany) and stored in cryoprotectant (25% ethylene glycol, 25% glycerol, and 0.05 mol/L phosphate buffer) at 4°C until use. Then, the brain sections were rinsed briefly in PBS and treated with 0.5% BSA for 30 min. The sections were incubated with rabbit anti-Iba1 (1:500 dilution, Wako) overnight at 4°C in the presence of 0.3% Triton X-100 and normal goat serum. They were then incubated for 2 h with an Alexa Fluor conjugated secondary antibody (diluted 1:500). Sections were finally washed in PBS and mounted using Vectashield mounting medium containing DAPI. The images were taken using a fluorescence microscope (Olympus Microscope System CKX53; Olympus, Tokyo, Japan). A threshold for positive staining was determined for each image that included all cell bodies and processes but excluded the background staining. Image quantification of mouse tissue region was performed using ImageJ 1.50i software (National Institutes of Health, Bethesda, MD, United States).

### Statistical Analyses

The data are expressed as the mean ± standard error of measurement. All of the experiments were performed at least three times. One-way analysis of variance was used to detect significant differences between the control and treatment groups. All statistical parameters were calculated using GraphPad Prism 7.0 software (GraphPad Software, San Diego, CA, United States). Dunnett’s test was used for multiple comparisons. The differences were considered significant at *P* < 0.05.

## Results

### Effect of TEC on the Viability in BV-2 Microglia

To exclude potential cytotoxicity of TEC, a CCK assay was performed. The BV-2 cells were treated with TEC for 24 h. TEC did not affect cell viability at doses ranging from 12.5 to 100 μM (**Figure 1B**). A non-toxic concentration (≤100 μM) was used in subsequent experiments. To ascertain the non-toxic concentration of the combination of TEC and LPS in BV-2 cells, cells were pretreated with TEC (25, 50, or 100 μM) for 2 h followed by treatment with LPS (1 μg/mL) for 22 h. Cell viability approached ≥ 90% under all experimental conditions, indicating no cytotoxicity of TEC in LPS-treated BV-2 cells (**Figure 1C**). These results suggest that TEC did not affect the viability of LPS-stimulated microglial cells.

### Effects of TEC on the Production of NO and PGE_2_ and the Expression of iNOS and COX-2 in LPS-Stimulated BV-2 Microglia

**FIGURE 2:**
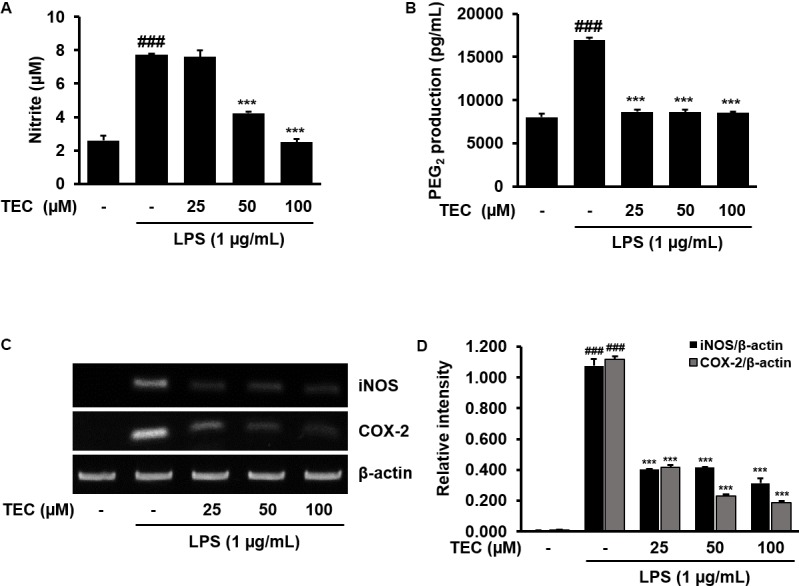
TEC inhibits the production and expression of inflammatory molecules in LPS-stimulated BV-2 microglia. Cells were pre-treated with TEC for 2 h followed by LPS stimulation (1 μg/mL) for 22 h. The supernatants are obtained and the amount of **(A)** nitrite and **(B)** PGE_2_ released into the media is determined. **(C)** Cells were pretreated with TEC for 1 h followed by LPS stimulation for 5 h and then RT-PCR was performed to see the effects of TEC on mRNA expression of iNOS and COX-2. **(D)** Bar graphs represent the relative expression of iNOS and COX-2 for **(C)**. The results are expressed as the mean ± SEM of three independent experiments. ^###^*P* < 0.001 vs. untreated control cells and ^∗∗∗^*P* < 0.001 vs. LPS-treated cells.

The potential inflammatory properties of TEC were evaluated against the production of two major inflammatory mediators, NO and PGE_2_, in LPS-stimulated BV-2 cells. To quantify the levels of NO and PGE_2_ production, the amounts of nitrite and PGE_2_ released into the culture medium were measured using Griess reagent and an ELISA, respectively. Based on the NO detection assay results, LPS alone markedly induced NO production compared to untreated controls (**Figure 2A**). However, pretreatment with TEC significantly reduced the level of NO production in LPS-stimulated BV-2 cells in a dose-dependent manner. Under the same conditions, stimulating the cells with LPS also resulted in a significant increase in PGE_2_ production; however, the effect of LPS on PGE_2_ production was markedly diminished by pretreatment with TEC (**Figure 2B**). RT-PCR analyses were conducted to determine whether the inhibition of NO and PGE_2_ production by TEC in LPS-stimulated BV-2 cells was associated with reduced levels of iNOS and COX-2 expression. As shown in **Figures 2C,D**, iNOS and COX-2 mRNA expression was markedly upregulated by treatment with LPS (1 μg/mL); however, TEC treatment attenuated iNOS and COX-2 expression in LPS-stimulated BV-2 cells.

### Effects of TEC on the Production of Inflammatory Cytokines and Expression of TNF-α and IL-6 in LPS-Stimulated BV-2 Microglia

**FIGURE 3:**
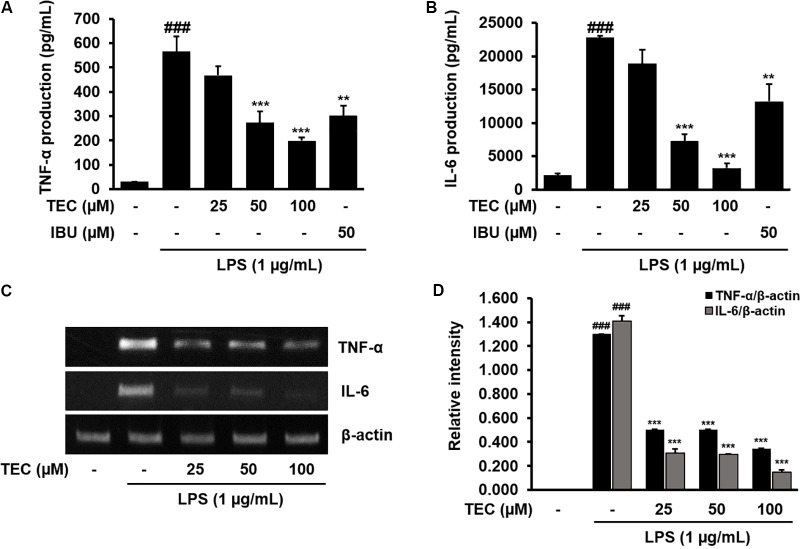
TEC inhibits the production expression of inflammatory cytokines in LPS-stimulated BV-2 microglia. Cells were pre-treated with TEC for 2 h followed by LPS stimulation (1 μg/mL) for 22 h. The supernatants are obtained and the amount of **(A)** TNF-α and **(B)** IL-6 released into the media is determined. **(C)** Cells were pretreated with TEC for 1 h followed by LPS stimulation for 5 h and then RT-PCR are performed to see the effects of TEC on mRNA expression of TNF-α and IL-6. **(D)** Bar graphs represent the relative expression of TNF-α and IL-6 for **(C)**. Ibuprofen (50 μM) was used as a positive control. The results are expressed as the mean ± SEM of three independent experiments. ^###^*P* < 0.001 vs. untreated control cells and ^∗∗^*P* < 0.01 or ^∗∗∗^*P* < 0.001 vs. LPS-treated cells.

The effects of TEC on the production of inflammatory cytokines, including TNF-α and IL-6, were analyzed using ELISA. BV-2 cells were incubated with various concentrations of TEC in the presence of LPS (1 μg/mL) for 24 h. As shown in **Figures 3A,B**, TNF-α and IL-6 levels were markedly increased in the culture media of LPS-stimulated BV-2 cells. However, pretreatment with TEC significantly reduced the LPS-mediated release of these inflammatory cytokines in a dose-dependent manner. Ibuprofen-treated cells were used as a positive control and exhibited significant decreases in TNF-α and IL-6 production in LPS-stimulated cells. In addition, the effects of TEC on LPS-stimulated TNF-α and IL-6 mRNA expression were studied using RT-PCR. As shown in **Figures 3C,D**, TNF-α and IL-6 mRNA levels of LPS-treated BV-2 cells were also decreased by TEC treatment, consistent with the results of the ELISA for the cytokines.

### Effects of TEC on NF-κB Activation in LPS-Stimulated BV-2 Microglia

**FIGURE 4:**
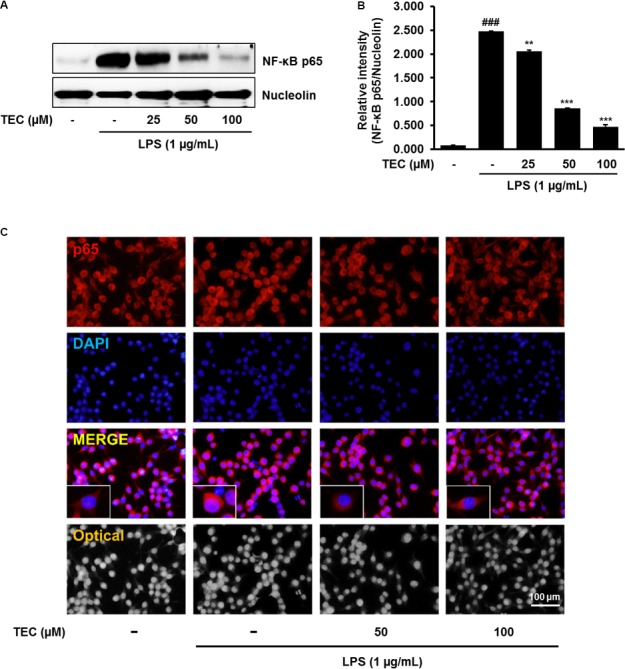
TEC suppresses the activation of NF-κB in LPS-stimulated BV-2 microglia. Cells were pre-treated with TEC for 1 h followed by LPS stimulation (1 μg/mL) for 30 min. **(A)** Nuclear translocation of NF-κB p65 was determined by western blot analysis. Nucleolin was use as a house keeping control gene for nuclear protein. **(B)** Bar graphs represent the relative expression of NF-κB p65 for **(A)**. ^###^*P* < 0.001 vs. untreated control cells and ^∗∗^*P* < 0.01 or ^∗∗∗^*P* < 0.001 vs. LPS-treated cells. **(C)** Translocation of NF-κB p65 into the nucleus was determined by Immunofluorescence analysis. Immunofluorescence microscope was used to detect the translocalization of NF-κB p65 (red) and nucleus (blue). Scale bar: 100 μm.

To investigate the molecular mechanisms underlying the anti-neuroinflammatory effects of TEC, the ability of TEC to prevent activation of NF-κB was examined by analyzing the nuclear translocation of NF-κB p65. NF-κB plays a major role in regulating a number of genes involved in inflammatory responses (Kempe et al., 2005). In the active state, NF-κB is released from IκBα through phosphorylation and degradation of IκBα, followed by translocation into the nucleus and regulation of proinflammatory gene expression (Kempe et al., 2005). Western blot analysis revealed that the amount of NF-κB p65 in the nucleus markedly increased in a dose-dependent manner following exposure to LPS; however, LPS-induced p65 levels in the nuclear fraction were reduced by TEC pretreatment (**Figures 4A,B**). Consistent with this, the results of immunostaining with an antibody against NF-κB p65 demonstrated that TEC inhibited nuclear translocation of the p65 subunit of NF-κB (**Figure 4C**).

### Effects of TEC on the Phosphorylation of MAPKs in LPS-Stimulated BV-2 Microglia

**FIGURE 5:**
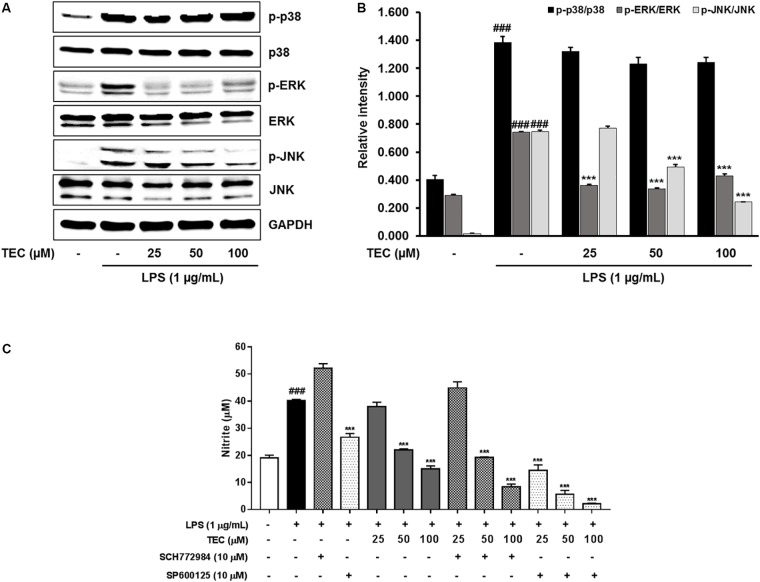
TEC suppresses the phosphorylation of MAPKs in LPS-stimulated BV-2 microglia. Cells were pre-treated with TEC for 1 h followed by LPS stimulation (1 μg/mL) for 30 min. **(A)** Western blot was performed to determine the effect of TEC on phosphorylation of MAPKs activity. **(B)** Bar graphs represent the relative expression of MAPKs phosphorylation for **(A)**. **(C)** Cells were pretreatment with TEC and inhibitors of p38 or JNK for 2 h and then stimulated with LPS (1 μg/mL) for 22 h and then nitrite was measured by Griess reagents. The results are expressed as the mean ± SEM of three independent experiments. ^###^*P* < 0.001 vs. untreated control cells and ^∗∗∗^*P* < 0.001 vs. LPS-treated cells.

To investigate whether TEC inhibits the production of inflammatory cytokines through the MAPK signaling pathway, we examined the effects of TEC on LPS-induced phosphorylation of p38, ERK, and JNK in BV-2 microglia by western blot analysis. As shown in **Figures 5A,B**, stimulation with LPS markedly increased the phosphorylation of p38 MAPK, ERK, and JNK. However, pretreatment with TEC markedly inhibited LPS-induced phosphorylation of ERK and JNK, but not p38 MAPK, compared to cells treated with LPS alone. These results suggest that the phosphorylation of ERK and JNK is involved in the inhibitory effects of TEC on LPS-induced inflammation in microglia. To determine if activation of either MAPK contributed to the anti-neuroinflammatory action of TEC, we used SCH772984, a selective inhibitor of ERK; and SP600125, a selective inhibitor of JNK. As shown in **Figure 5C**, SCH772984 and SP600125 inhibited TEC-induced NO production in a dose-dependent manner, implying that they play a role in LPS-induced inflammation.

### Effect of TEC on Microglial Activation and the Production of Inflammatory Action in LPS-Stimulated Mice

**FIGURE 6:**
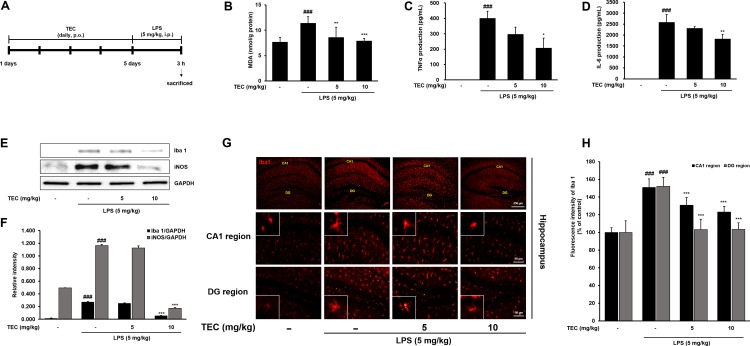
TEC inhibits neuroinflammation in LPS-stimulated mice. **(A)** Mice were assigned to four groups (*n* = 7/group) and administered vehicle (saline) or TEC at doses of 5 or 10 mg/kg orally for 5 days after adaptation for 1 week. On day 5, LPS (5 mg/kg) were administered 3 h before the sacrificed. **(B)** MDA levels were measured in hippocampus of the mouse model. **(C,D)** Production of TNF-α **(C)** and IL-6 **(D)** in the serum were measured using ELISA. **(E)** The expression of Iba1 and iNOS was detected by western blotting using specific antibodies in murin hippocampus tissues. GAPDH protein was used as an internal control. **(F)** Bar graphs represent the relative expression of Iba1 and iNOS for **(E)**. **(G)** Immunoreactive cells of anti-Iba1 antibody was investigated two different region (CA1; cornu ammonis 1 and DG; dentate gyrus) in the brain hippocampus by immunofluorescence analysis. **(H)** Bar graphs represent the fluorescence intensity of Iba1 for **(G)**. ^###^*P* < 0.001 vs. untreated mice and ^∗^*P* < 0.05, ^∗∗^*P* < 0.01, or ^∗∗∗^*P* < 0.001 vs. LPS-treated mice.

To further examine the inhibitory effects of TEC on inflammatory responses identified *in vitro*, LPS treatment in a murine model of inflammation was used to study the suppressive effects of TEC on neuroinflammation *in vivo*. The MDA level increased significantly in the LPS-stimulated group (*p* < 0.001) in hippocampal tissue. However, the MDA level in the LPS-stimulated group was significantly decreased by treatment with TEC (5 or 10 mg/kg) (**Figure 6B**). As shown in **Figures 6C,D**, TNF-α and IL-6 levels in serum were significantly increased in LPS-injected mice compared to control mice. However, administration of TEC significantly inhibited TNF-α and IL-6 release in serum compared to LPS-administered mice, consistent with the *in vitro* results. Furthermore, we conducted immunohistochemistry and western blotting to detect the expression of Iba-1, a marker of microglial activation (Kim et al., 2017) in the brain. LPS-treated mice demonstrated increased expression of Iba1-positive cells compared to vehicle-treated controls; however, expression of Iba-1 was decreased in TEC-treated mice (**Figures 6E–H**). In addition, as shown in **Figures 6E,F**, protein expression of iNOS in the brain was significantly up-regulated in response to LPS. In contrast, TEC inhibited iNOS protein expression in LPS-induced mice.

### Effects of TEC on TLR4 and MyD88 Activation in LPS-Stimulated BV-2 Microglial and Mouse Models

**FIGURE 7:**
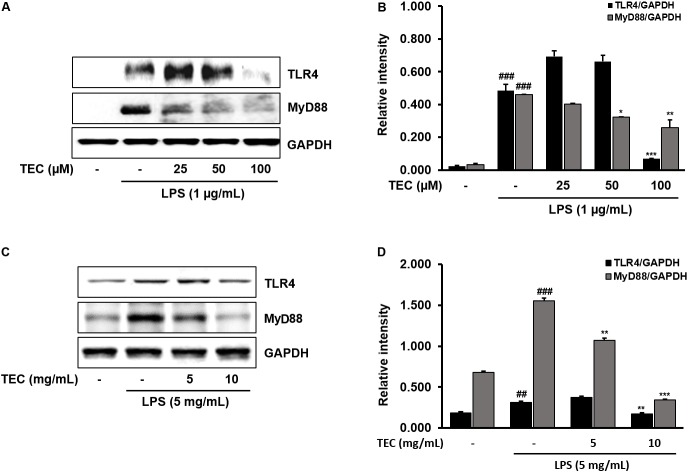
TEC suppresses the expression of TLR4 and MyD88 in LPS-stimulated BV-2 cells and mice. **(A,C)** Western blotting analysis was performed to quantify the TLR4 and MyD-88 in BV-2 cells and murin hippocampus tissues. **(B,D)** Band intensities were quantified by densitometric analysis and normalized by GAPDH. ^##^*P* < 0.01 or ^###^*P* < 0.001 vs. untreated cells and mice and ^∗^*P* < 0.05, ^∗∗^*P* < 0.01, or ^∗∗∗^*P* < 0.001 vs. LPS-treated cells and mice, respectively.

TLR4 is an important receptor of LPS and interacts with adaptor molecules such as MyD88 that are critical for the activation of downstream inflammation-related signaling pathways (Zhang et al., 2014). We investigated the effect of TEC on activation of TLR4 and MyD88 in LPS-induced BV-2 cells and *in vivo* in mice. As shown in **Figures 7A,B**, TLR4 and MyD88 protein expression was significantly increased in LPS-treated cells compared to non-treated cells. However, TEC reduced the protein expression of TLR4 and MyD88 compared to LPS-treated cells. Similarly, TLR4 and MyD88 protein expression in LPS-treated mice was markedly increased compared to the untreated group. In contrast, pre-treatment with TEC inhibited LPS-induced TLR4 and MyD88 protein expression. These results suggest that the anti-neuroinflammatory action of TEC in LPS-induced BV-2 cells and mice is associated with modulation of TLR4 and MyD88 pathways (**Figures 7C,D**).

## Discussion

Natural products are promising sources of therapeutic molecules that have the potential to be developed as treatments for neurodegenerative disorders (Balunas and Kinghorn, 2005). The ability of dietary phytochemicals to regulate cellular stress responses points to novel therapeutic targets for neurodegenerative disorders. In this study, we assessed the flavonoid TEC as a potential therapeutic for neurodegenerative diseases.

TEC is an *O*-methylated isoflavone, a type of flavonoid, isolated from leopard lily *Belamcanda chinensis*, *Iris unguicularis*, or *Pueraria thunbergiana* (Lee et al., 2001; Thelen et al., 2005; Atta-Ur-Rahman et al., 2010). TEC has attracted considerable attention due to its known biological actions, such as antitumor effects (Wani et al., 2013), antibacterial properties (Oh et al., 2001), and free radical neutralization (Kang et al., 2005). Although Pan et al. reported an anti-inflammatory action of TEC (Pan et al., 2008), this effect was demonstrated using macrophages and not glia. Moreover, the regulatory mechanisms underlying TEC-induced effects in neurodegenerative diseases such as AD have yet to be elucidated. In neurodegenerative diseases, inflammation may be triggered by the accumulation of proteins with abnormal conformations or by signals emanating from injured neurons (Hong et al., 2016). Suppression of neuroinflammation is considered an important therapeutic goal. Therefore, inhibition of microglial activation may be a promising strategy for treating neurodegenerative diseases. The present investigation sought to examine the inhibitory effects of TEC on neuronal inflammation using both *in vitro* and *in vivo* experimental models. We used BV-2 cells (immortalized murine microglia) and induced an inflammatory reaction by stimulating microglia with LPS. In response to inflammatory stimuli such as LPS, microglial activation promotes the production of inflammatory mediators such as NO and PGE_2_. These characteristic neuroinflammatory processes induce neuronal damage and can subsequently lead to neurodegenerative disorders (Amor et al., 2010). Accumulating evidence also supports a role for ROS in triggering microglial activation, which regulates a variety of inflammatory factors such as NO and PGE_2_.

We examined the effect of TEC on ROS generation in LPS-stimulated BV-2 microglial cells. Fluorometric data showed that LPS treatment significantly increased the generation of ROS generation, whereas treatment with TEC significantly decreased LPS-induced generation of ROS in a dose-dependent manner. The inhibitory effects of TEC and *N*-acetyl-L-cysteine (NAC) were comparable, suggesting that TEC may act as an ROS inhibitor (data not shown). In the central nervous system (CNS), accumulation of ROS triggers activation of microglia and further exacerbates neuroinflammatory responses, leading to the progression of neurodegenerative disorders (Uttara et al., 2009; Hsieh and Yang, 2013). The production of ROS can induce aberrant production of proinflammatory factors such as NO and PGE_2_ in microglia through activation of various downstream signaling mediators, such as NF-κB and MAPK (Park et al., 2015). To elucidate the molecular mechanisms responsible for the neuroinflammatory effects of TEC, we further investigated the inhibitory effects of TEC on the production of inflammatory factors induced by LPS in BV-2 microglial cells. Our results demonstrate that TEC significantly inhibited LPS-induced microglial activation and the production of NO, PGE_2_, TNF-α, and IL-6. Consistent with these findings, TEC also significantly attenuated LPS-induced mRNA expression of iNOS, COX-2, TNF-α, and IL-6. These results provide support for the potential therapeutic effects of TEC in various inflammatory responses.

NF-κB plays an important role in the regulation of microglia-mediated neuroinflammation. However, dysregulation of NF-κB has been linked to aberrant neuroinflammation by the upregulation of proinflammatory mediators (Maqbool et al., 2013). Indeed, NF-κB-binding specific regions have been identified in proinflammatory genes such as iNOS, COX-2, and TNF-α (Kempe et al., 2005). Therefore, inhibition of NF-κB transcriptional activity in microglia may regulate the progression of neurodegenerative diseases caused by neuroinflammation. As such, it has become a molecular target of interest for therapeutic anti-inflammatory agents (Camandola and Mattson, 2007). We showed that LPS stimulation resulted in NF-κB translocation from the cytosol to the nucleus, whereas TEC inhibited LPS-mediated nuclear translocation of NF-κB in BV-2 cells. These findings indicate that the inactivation of NF-κB at least partly underlies the anti-inflammatory effect of TEC in LPS-stimulated BV-2 cells.

MAPKs, a family of serine/threonine protein kinases including p38, ERK, and JNK, play a crucial role in controlling signaling events that contribute to the production of neuroinflammatory mediators (Wang et al., 2004; Velagapudi et al., 2014). Previous studies have demonstrated the significance of MAPKs in the transcriptional regulation of LPS-induced production of inflammatory mediators (Song et al., 2012; Jung et al., 2016). In our experiment, pretreatment with TEC decreased phosphorylated MAPKs, especially phosphorylated ERK and JNK, without a change in the total levels of MAPKs. In addition, we examined whether the effect of TEC on LPS-stimulated production of inflammatory mediators was achieved by suppression of ERK and JNK activation. Here, we showed that cotreatment with TEC and the ERK inhibitor SCH772984 or the JNK inhibitor SP600125 further suppressed the production of NO compared to TEC alone in LPS-stimulated BV-2 cells. Thus, the suppressive effect of TEC on ERK and JNK activation appears to be achieved by inhibition of inflammatory responses.

We used an *in vivo* mouse model of acute brain inflammation induced by systemic LPS administration to further examine the inhibitory effects of TEC on neuroinflammation. Microglia are known to be activated in response to brain injuries and immunological stimuli, upon which they undergo dramatic alterations in morphology, changing from resting, ramified microglia into activated microglia (Park et al., 2012). Iba-1 is a microglia/macrophage-specific calcium-binding protein (Imai et al., 1996). Iba-1 plays important roles in the regulation of several immunological and pathophysiological functions of microglia and serves as a unique marker for detecting microglial activation. Our immunofluorescence assays against Iba-1 found that TEC administration diminished the morphological changes indicative of an activated form that can manifest as increased cell size and irregularly shaped processes in the hippocampal CA1 and dentate gyrus regions. Moreover, immunoblotting analysis revealed that TEC administration significantly reduced the Iba-1 protein expression enhanced by LPS injection in the brain compared to LPS-treated control mice. In addition, the antioxidative activity of TEC was accompanied by reduction of MDA and iNOS levels. TEC also significantly inhibited TNF-α and IL-6 release in serum compared to LPS-treated controls. These effects were consistent with those observed in our *in vitro* experiments.

TLR4 is activated by LPS-mediated inflammation in microglia, and is also found in the CNS where it regulates neurogenesis (Rolls et al., 2007; Seong et al., 2016). TLR4 is activated via interaction with MyD88. TLR4-MyD88-mediated signaling promotes activation of NF-κB and MAPK signaling, leading to the production of inflammatory mediators (Ko et al., 2016; Seong et al., 2016). Therefore, we detected the effects of TEC on LPS-induced TLR4 and MyD88 expression in BV-2 cells and murine hippocampal tissue. The results showed that TEC inhibited LPS-induced TLR4 and MyD88 expression, suggesting that TEC exhibited its neuroinflammatory effects by modulating TLR4-MyD88 interaction.

## Conclusion

**FIGURE 8:**
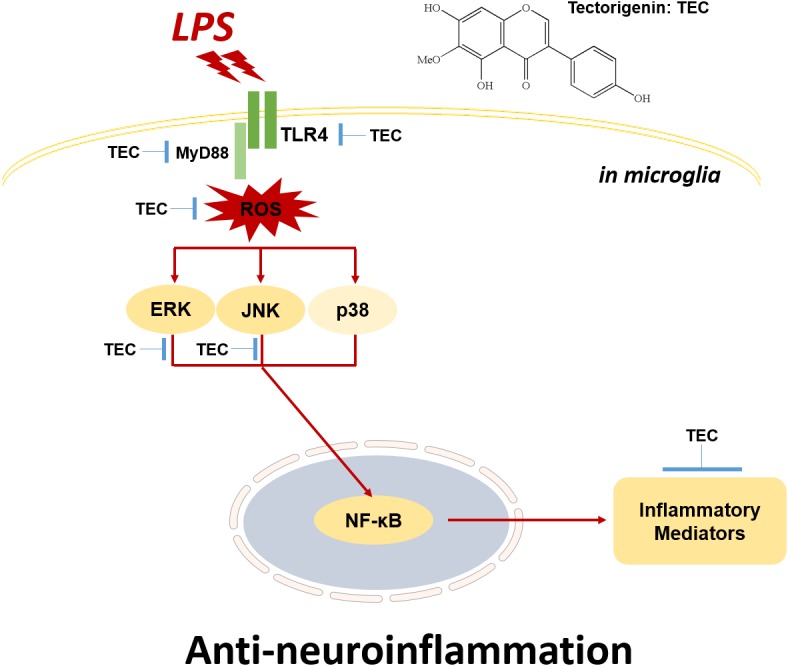
Schematic model of the neuroinflammatory modulation afforded by TEC in activated microglia. TEC inhibits LPS-stimulated neuroinflammation through TLR4-MyD88-mediated NF-κB and ERK/JNK pathways.

TEC effectively protected against LPS-induced damage, oxidative stress, and inflammation. Notably, these effects were corroborated both *in vitro* and *in vivo*. These beneficial effects may be the result of suppression of ROS generation; decrease in NO, PGE_2_, TNF-α, and IL-6 secretion; and inhibition of iNOS, COX-2, TNF-α, and IL-6 expression. Moreover, TEC significantly decreased activated microglial cells, TNF-α, and IL-6 levels in LPS-stimulated mice. These mechanisms may be closely associated with TLR4-MyD88-mediated inhibition of ERK/JNK and NF-κB (**Figure 8**). The components of these pathways may, therefore, represent potential targets for the prevention and treatment of neuroinflammatory disorders. Furthermore, searching for phytochemicals with both anti-inflammatory and antioxidative activities may be a promising approach for the treatment of various neurodegenerative diseases.

## Author Contributions

H-SL and S-JJ conceived and designed the experiments. H-SL, YK, B-YK, GP, and S-JJ performed the experiments. H-SL and S-JJ conduced to the cell experiments. H-SL, YK, GP, and S-JJ assisted with the animal experiments. H-SL, B-YK, and S-JJ analyzed the data and performed the statistical analysis. H-SL and S-JJ wrote and helped to modify the paper. All authors read and approved the final version of the manuscript.

## Conflict of Interest Statement

GP was employed by company Ektos Industries Co., Ltd. The other authors declare that the research was conducted in the absence of any commercial or financial relationships that could be construed as a potential conflict of interest.
